# Spontaneous and uncomplicated anal elimination of a hip pin—a case report

**DOI:** 10.3109/17453674.2012.718519

**Published:** 2012-08-25

**Authors:** Helge Wangen, Lars Hemstad, Håkan Jonsson, Ove Talsnes

**Affiliations:** ^1^Department of Orthopaedic Surgery; ^2^Department of Radiology, Innlandet Hospital Trust, Elverum, Norway.

In 2005 a 78-year-old woman was admitted with an undisplaced proximal femoral fracture and was operated with osteosynthesis by 2 hip pins (Smith and Nephew, Tuttlingen, Germany). Postoperatively, she was allowed full weight bearing and left hospital 14 days later. At first follow-up, 8 weeks after surgery, the patient walked with an ambulatory device and admitted that she had some pain. The radiographs showed a displaced fracture (Figure 1A-C). The patient was informed about possible prognosis outcome, but she was satisfied with the progress thus far and refused any kind of further treatment at this point.

**Figure 1.**
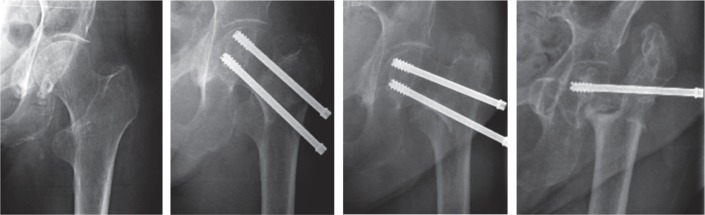
A. Undislocated primary fracture. B. The day after surgery, showing dislocation. C. Two months postoperatively. D. Six years postoperatively.

Follow-ups at 5 and 8 months revealed further displacement of an unhealed fracture. The patient was still not motivated to have further treatment, and no additional follow-up was planned.

In June 2011, the patient felt moderate anal discomfort. During defecation the following day, she experienced pain and needed aid from her home-based nurse to extract a foreign body which slipped into the toilet bowl with a metallic sound. With suspicions about the origin of the screw, it was presented to the patient’s GP and she was admitted to our outpatient clinic for further investigation.

The patient was completely asymptomatic in the abdomen, and her anal discomfort had disappeared. The radiograph (Figure 1D) revealed only 1 remaining hip pin and the screw presented to us was confirmed to be identical to the missing distal screw implanted in the patient’s left hip 6 years earlier. The fracture was greatly displaced, with no sign of healing and the remaining screw was still in situ; it was displaced, but without any major migration. The skin showed no recent scars or signs of perforation.

To avoid further complications, including pelvic migration, the remaining screw was easily removed percutaneously under local anesthesia. In order to reveal any possible intrapelvic damage, and at the same time hoping to track the pathway of the migrated screw, we performed a CT scan (Figure 2A-C) with 3D reconstructions (Figure 3A and B). The CT scans left no doubt regarding the pathway of the screw. The femoral head was perforated with a canal similar to the diameter of the screw and there was a crater in the pelvic bone located in line with the perforation through the femoral head. There was no sign of bowel perforation, but some scar tissue was located between the rectum and the crater surrounding the pelvic perforation. The location of the scar tissue and the pelvic bone perforation indicated a migration into the extraperitoneal part of the colon.

**Figure 2.**
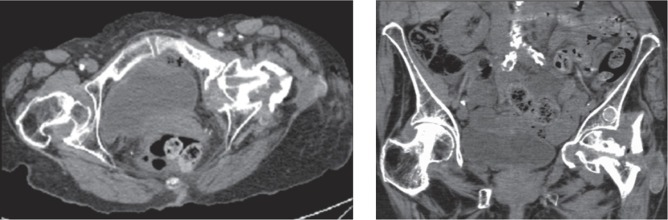
CT scans showing the canal through which the hip pin had migrated through the femoral head and the pelvic bone before entering the abdomen.

**Figure 3.**
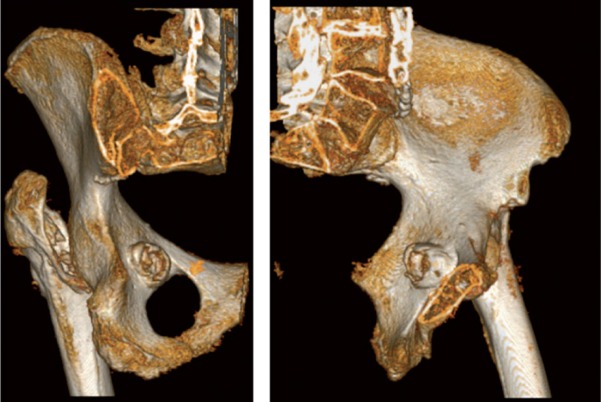
CT reconstruction (3D) of the entry hole through the pelvic bone, identifying the entry point of the screw to the abdomen before entering the rectum on its way to defecation.

## Discussion

Intrapelvic migration of metallic implants from osteosynthesis of hip and pelvic fractures is a well known—but still rare—complication (Kottmeier et al. 1993, Carstensen and Noer 1998, Kircher et al. 2005, Sayegh et al. 2005, Tauber and Resch 2006, Bhatti and Abbasi 2007, Heineman et al. 2010, Lucke et al. 2010). The frequency of this complication was described by Adolphson (1995) who found intrapelvic migration of one or more hip fracture screws in 7 of 1,307 patients, approximately 0.5% of the cases. Migrated screws were removed surgically and none of the cases were fatal. One fatality was described by Sundgren and Persson (1994); this was due to an iliac artery injury. Manufacturers of orthopedic implants and surgeons are aware of this potentially fatal complication, and the design of hip pins and screws has been adjusted in order to prevent migration (Olerud et al. 1995), e.g. screws with a collar larger in diameter than the shaft. The screw used in our case had such a design, but clearly this did not prevent pelvic migration.

The rectum is a retroperitoneal part of the bowel. The screw may have passed into the rectum without entering the abdominal cavity. Such a passage would bypass the abdominal cavity, leaving the peritoneum unaffected and avoiding leakage from the intestine into the abdominal cavity. This could explain the rather surprising lack of abdominal discomfort, pain, and peritonitis. This mechanism resembles the one observed when a stone from the gall bladder penetrates the duodenum and finally causes ilieus without a previous peritonitis.

Undisplaced proximal femoral fractures and fractures in young patients are often reduced and fixed with screws. In our case, the main reason for the early displacement was probably the choice of fixation. The radiographs (Figure 1A-C) show an undisplaced trochanteric fracture rather than an intracapsular fracture.

Our patient was followed rigorously and was offered treatment for her complication, but she refused treatment initially and has still not accepted our offer of a total hip arthroplasty.

## References

[CIT0001] Adolphson P (1995). Intrapelvic penetration of Olmed cervical hip fracture screws. A report of 7 cases. Acta Orthop Scand.

[CIT0002] Bhatti A, Abbasi A (2007). Intra pelvic total migration of sliding screw in intertrochanteric fracture. J Coll Physicians Surg Pak.

[CIT0003] Carstensen JP, Noer HH (1998). Medial migration of a Hansson hook-pin after femoral neck fracture. Ugeskr Laeger.

[CIT0004] Heineman DJ, van Buijtenen JM, Heuff G, Derksen EJ, Poll RG (2010). Intra-abdominal migration of a lag screw in gamma nailing: report of a case. J Orthop Trauma.

[CIT0005] Kircher J, Durr HR, Jansson V (2005). Intrapelvic pin migration after periacetabular reconstruction and arthroplasty of the hip in metastatic pelvic disease--a case report. Acta Orthop.

[CIT0006] Kottmeier S, Born CT, Saul H (1993). Laparoscopic retrieval of a migrating intrapelvic pin: case report and review of the literature. J Trauma.

[CIT0007] Lucke M, Burghardt RD, Siebenlist S, Ganslmeier A, Stockle U (2010). Medial migration of lag screw with intrapelvic dislocation in gamma nailing--a unique problem? A report of 2 cases. J Orthop Trauma.

[CIT0008] Olerud S, Olerud C, Rehnberg L (1995). A screw for cervical hip fractures designed to minimize migration. Acta Orthop Scand.

[CIT0009] Sayegh FE, Tsintzas D, Kapetanos GA (2005). Intrapelvic migration of a guide pin during fixation of a hip fracture: who and what is to blame?. Acta Orthop Belg.

[CIT0010] Sundgren K, Persson L (1994). Penetrating cervical hip fracture screws. Report of 4 cases. Acta Orthop Scand.

[CIT0011] Tauber M, Resch H (2006). Sigmoid perforation after medial migration of lag screw in gamma nailing. Arch Orthop Trauma Surg.

